# HER2 expression and efficacy of T-DM1

**DOI:** 10.1186/s13058-014-0478-7

**Published:** 2014-12-03

**Authors:** Filippo Montemurro

**Affiliations:** Unit of Investigative Clinical Oncology (INCO), Fondazione del Piemonte per l’Oncologia, Candiolo Cancer Institute (IRCCs), Strada Provinciale 142, Km 3.95, Candiolo, 10060 Italy

I have read with great interest the article by Edith Perez and colleagues reporting a correlation between trastuzumab–emtansine (T-DM1) efficacy and human epidermal receptor 2 (HER2) mRNA expression in the TDM4450g, phase II randomized clinical trial [1]. While acknowledging that these results are merely hypothesis generating, I believe some findings from this analysis deserve further reasoning.

Upon central histopathological review, the proportion of patients whose tumors had a normal HER2 status was evenly distributed in the two arms of the trial (about 14%, see Table one in [1]). However, and not surprisingly, these HER2-normal tumors clustered entirely in the low HER2 mRNA group (Table two in [1]), where they accounted for slightly less than one-third of the cases. At the same time, the proportion of tumors with hormone receptor expression was higher in the low HER2 mRNA group (62.1% vs. 46.6%). These data indicate that, beyond the number of HER2 receptors on the cell surface suitable for T-DM1 binding, mRNA HER2 levels are also related to different intrinsic biology and, possibly, reliance not only on HER2 but also upon other regulatory pathways such as hormone-receptor signaling [2].

In other terms, the efficacy of T-DM1 over docetaxel–trastuzumab was compared in two biologically different groups of patients, one of which was enriched with true HER2-positive tumors, and the other being a mix of HER2-negative and HER2-positive tumors at the lower end of the spectrum to define HER2-positivity. I therefore believe the extremely interesting finding of this analysis is the comparable activity of T-DM1 and docetaxel–trastuzumab in the low mRNA HER2 group, paralleled with reduced toxicity. Indeed, a combined rate of response of 11% was reported in the TDM4258 and TDM437g phase II trials in 36 heavily pretreated women whose tumor turned out to be HER2 normal by central reanalysis [3,4].

Should an activity of T-DM1 comparable with that of conventional chemotherapy be confirmed in tumors with low HER2 expression, even in those not fulfilling the conventional criteria for HER2 positivity, this would translate into an increased number of patients benefiting from this agent that, by virtue of its mechanism of action, has shown to be excellently tolerated.

## Abbreviations

HER2: Human epidermal receptor 2
TDM-1: Trastuzumab–emtansine
